# Gastric Cancer Literacy and Related Factors in Iran: A Cross‐Sectional Study

**DOI:** 10.1002/cnr2.70486

**Published:** 2026-04-28

**Authors:** Fatemeh Pourhaji, Mohammad Hossein Delshad, Kianoosh Yavarmanesh

**Affiliations:** ^1^ Department of Public Health Department, School of Health Torbat Heydariyeh University of Medical Sciences Torbat Heydariyeh Iran; ^2^ Health Sciences Research Center Torbat Heydariyeh University of Medical Sciences Torbat Heydariyeh Iran; ^3^ Department of Medicine, School of Medicine Gonabad University of Medical Sciences Gonabad Iran; ^4^ Students Research Committee, Faculty of Medicine Gonabad University of Medical Sciences Gonabad Iran

**Keywords:** attitude, gastric cancer, health literacy, knowledge, screening

## Abstract

**Background:**

Gastric cancer (GC) is one of the most prevalent and deadly cancers worldwide, particularly in countries like Iran. Gastric cancer health literacy (GCHL) plays a key role in early detection, prevention, and timely treatment. This study aims to investigate the level of GCHL and its associated factors among adults in Torbat Heydarieh, northeastern Iran.

**Methods:**

In this cross‐sectional study conducted from January 5, 2022, to September 26, 2023, 304 clients from Comprehensive Health Service Centers (CHSCs) in Torbat Heydarieh were surveyed. Data were collected using a validated tool that measured sociodemographic characteristics and Gastric cancer health literacy questionnaire (GCHLQ), knowledge of risk factors, symptoms, prevention methods, and screening, and attitude questionnaire included beliefs about GC prevention, curability at early stages, the benefits of early detection, previous screening history, and reasons for not undergoing screening. Data were analyzed using SPSS software version 25 using descriptive statistics, correlation tests, and multiple linear regression to determine predictors of GCHL. The statistical significance level was set at *p* < 0.05.

**Results:**

Participants demonstrated a moderate (31–39) level of GCHL. Higher GCHL scores were significantly associated with being female, having a higher educational level, and a family history of GC. The multivariate regression model explained 51.4% of the variance in GCHL. In this model, family history of GC, knowledge score, and female gender were significantly associated with higher GCHL scores.

**Conclusions:**

The findings emphasize the importance of targeted educational and awareness programs to improve GCHL, particularly among older adults and rural populations. Future longitudinal and qualitative studies are recommended to better understand barriers to cancer literacy and improve health outcomes through informed interventions.

**Abbreviations**
ASIR: age‐standardized incidence rate
ASMR: age‐standardized mortality rate
CHSCs: comprehensive health service centers
CS: cancer screening
GBD: global burden of diseases
GC: gastric cancer
GCHL: gastric cancer health literacy
GCO: global cancer observatory
GCS: gastric cancer screening
HBM: health belief model
HL: health literacy
*H. pylori*
:
*Helicobacter pylori*

NGOs: non‐governmental organizations
PHC: primary health care
TPB: theory of planned behavior
WHO: World Health Organization

## 1 Background

Gastric cancer (GC) is the fifth most common malignancy and the fourth leading cause of cancer‐related death worldwide, with an estimated 769 000 deaths in 2020 [1, 2]. Despite advances in diagnosis and treatment, GC remains a major public health challenge, particularly in regions with high incidence [3] such as East Asia, where age‐standardized incidence rates (ASIR) reach 32.5 per 100 000 in men and 13.2 in women [4]. These elevated rates are influenced by dietary habits, high prevalence of *Helicobacter pylori* infection, and genetic factors. In contrast, lower incidence is observed in regions like Middle Africa and parts of North America, likely due to differences in dietary patterns, lower *H. pylori* prevalence, or limited access to diagnostic services, potentially leading to underdiagnosis and underreporting [5, 6]. In Iran, GC consistently ranks among the leading causes of cancer‐related mortality. According to GLOBOCAN 2020 and national data, it was the deadliest cancer in 2018, with an estimated age‐standardized mortality rate (ASMR) of 94 per 100 000 [4], and an age‐standardized incidence rate (ASIR) of 17.5 per 100 000 [7]. More recent findings from the Global Burden of Diseases (GBD) project indicate that between 2011 to 2021, GC became the tenth leading cause of cancer‐related deaths in Iran, reflecting a concerning upward trend [4]. Given the high mortality and delayed diagnosis, one key factor influencing early detection is health literacy (HL).

HL—the ability to access, understand, and use health information to make informed health decisions [8]—is a critical factor in cancer prevention [5, 9]. Inadequate HL can delay the recognition of early GC symptoms, reduce participation in screening programs, and hinder treatment adherence, particularly concerning in Iran, where public knowledge of *H. pylori*, dietary risk factors, and early warning signs remains limited [7, 10, 11, 12]. Furthermore, cultural beliefs and limited access to the health system contribute to disparities [13].

Effective participation in cancer screening programs, including endoscopy, *H. pylori* testing, and nutritional counseling, requires adequate HL. In Iran, there are no complementary screening services, and such services are not consistently integrated into national programs, particularly for high‐risk or underserved populations [14]. In this context, a formal, comprehensive population‐based GCS program is absent, meaning that individuals are not systematically invited or screened at the national level. Screening for GC in Iran is “opportunistic screening”, meaning it is performed only when a person has symptoms or when a physician recommends testing due to family history or other risk factors, rather than as part of an organized program for the general population [15].

In Iran, CHCs are key components of the Primary Health Care (PHC) system, designed to provide integrated, accessible, and preventive health services to the population at the community level [16].

HL also serves as a connecting factor between an individual's education, cultural background, environment, and healthcare experience [8]. Studies have associated inadequate HL with poorer health outcomes, higher medical costs [17], and reduced engagement in preventive services, including cancer screening [17, 18, 19, 20, 21, 22, 23, 24].

According to Oldach and Katz [7], limited HL is likely linked to poor cancer screening (CS) behavior, though further research is needed to establish this connection more clearly.

In northern Iran, Mansour‐Ghanaei et al. [25] reported low public awareness of GC symptoms, risk factors, and prevention strategies. Despite the high burden of GC in Iran, few studies have explored how HL affects early detection and prevention, particularly in high‐incidence regions. This gap in understanding the population's knowledge, attitudes, and risk perception represents a missed opportunity for timely intervention. As most GC cases are detected at later stages, enhancing public awareness of early warning signs, including persistent indigestion, abdominal pain, loss of appetite, unexplained weight loss, early satiety, fatigue, nausea or vomiting (possibly with blood), black or bloody stool, and bloating, and risk factors is essential.

Given the high prevalence of GC in Iran and region‐specific factors such as widespread *H. pylori* infection and distinctive habits, enhancing HL is particularly critical for effective prevention and early detection [26]. Low levels of public knowledge and culturally rooted beliefs highlight the urgent need for interventions to improve preventive behaviors and increase participation in screening programs [27]. Considering these barriers and gaps in understanding, it is crucial to explore the specific regional context more comprehensively.

To address this gap, the present study was conducted to assess gastric cancer health literacy (GCHL) and its associated factors among clients of CHCs in Torbat Heydarieh, a region with relatively high GC prevalence. These centers serve as key providers of integrated preventive, educational, and clinical services at the community level under the Ministry of Health and Medical Education.

GCHL is a multidimensional construct that includes individuals' ability to access, understand, and apply health‐related information. It also involves their knowledge and attitudes toward cancer prevention. Assessing knowledge and attitudes alongside is grounded in established health behavior theories such as the Health Belief Model (HBM) and the Theory of Planned Behavior (TPB). These frameworks emphasize how individuals' knowledge and attitudes influence their engagement in preventive behaviors. While GCHL provides a general measure of individuals' ability to process and act on health information, it may not fully capture cognitive or emotional factors that influence health decision‐making. Therefore, evaluating knowledge and attitudes in parallel with GCHL offers a more comprehensive understanding of the factors influencing preventive behaviors and supports the development of effective, culturally sensitive interventions. This study also explores the interrelationship between GCHL, knowledge, and attitudes. Understanding these interrelated components is crucial for designing culturally tailored educational interventions that enhance early detection and help reduce the GC burden in Iran.

## 2 Methods

This cross‐sectional study was conducted from January 5, 2022, to September 26, 2023, and included 304 clients from Comprehensive Health Service Centers (CHSCs) in Torbat Heydarieh, northeastern Iran. CHSCs are key components of the extensive Primary Health Care (PHC) network aiming for universal health coverage by providing preventive care, integrated services, including maternal and child health, non‐communicable disease management, immunization, and health education [16].

A two‐stage cluster sampling method was employed. In the first stage, 19 CHSCs were randomly selected as clusters. In the second stage, participants from each selected center were chosen using simple random sampling based on the national identification ID codes available in the SINA electronic health system (an online electronic health record system in Iran).

Inclusion criteria were as follows: age ≥ 30 years (consistent with the Iranian Non‐communicable Disease Risk Assessment Program, which targets individuals aged 30 years and above) [28], having an active medical record at the study center, providing written informed consent, possessing basic literacy sufficient to complete the questionnaire independently, and absence of self‐report or medically documented cognitive impairments that could interfere with study participation. Exclusion criteria were applied after initial eligibility screening and included: (1) participant withdrawal at any stage of data collection, (2) questionnaires with missing data exceeding the pre‐specified threshold for key variables (e.g., > 20% missing items), and (3) newly identified or previously undiagnosed serious mental disorders that could compromise data validity, as documented in medical records during the study period. Notably, individuals with a current or past diagnosis of GC were not eligible for inclusion in the analytic sample, as the study focuses on primary prevention and early detection in the general at‐risk population. Medical records were reviewed to confirm the absence of such diagnoses prior to enrollment. If any such cases were inadvertently included, they were excluded from all primary analyses.

Due to the lack of prior data on GCHL in the population, the sample size was calculated based on a conservative estimate with a prevalence of 50% (*p* = 0.5), a 95% confidence level (*Z* = 1.96), and a margin of error of 6.5% (*d* = 0.065). Considering a design effect (DEFF) of 1.2, the required sample size was estimated to be approximately 304 participants.

The standard formula for calculating the initial sample size for estimating a proportion is

$$n_{0}=\frac{Z^{2}\times p\times \left(1-p\right)}{d^{2}}$$

n_0 =_ initial sample size (before design effect), *Z* = *Z*‐value corresponding to the desired confidence level (1.96 for 95%), and *P* = estimated prevalence or proportion (0.5 used as a conservative estimate).

$$n_{0}=\frac{{\left(1.96\right)}^{2}.0.5.\left(1-0.5\right)}{{\left(0.065\right)}^{2}}=\frac{3.814.0.25}{0.004225}=\frac{0.9604}{0.004225}\approx 227.3$$

So, the unadjusted required sample size is approximately 227 participants. Because cluster sampling was used, the sample size must be adjusted:

$$n=n_{0}.\text{DEFF}=227.3.1.2=272.8$$

when rounded, this gives approximately 273 participants. To account for potential non‐response or incomplete data, a buffer, which is typically between 10% and15%, is added. The final sample size ≈273 + (10% buffer) ≈300–304 participants.

The research instrument included: (1) sociodemographic characteristics such as age, gender, marital status, educational level, occupational status, and family history of GC; (2) a validated knowledge and attitude questionnaire about GC (2), and (3) a researcher‐developed GCHLQ.

While knowledge and attitude reflect static awareness and beliefs about GC, GCHL represents a dynamic, action‐oriented capacity grounded of HL. GCHL encompasses not only the ability to access and understand health information but also to critically appraise it and apply it in decision‐making extending beyond factual recall or favorable opinions [29].

The knowledge questionnaire consisted of 22 items covering various aspects of GC, including its etiology, risk factors, symptoms, prevention methods, awareness of diagnostic tests such as endoscopy, and management (diagnosis and treatment). Responses were rated on a three‐point scale: 1 = “True”, 2 = “False”, 3 = “I don't know.” Each correct answer was awarded one point, resulting in a total score ranging from 0 to 22 knowledge levels were categorized as follows: high knowledge (scores of 15–22), moderate knowledge (scores of 8–14), low knowledge (scores 0–7) [14].

The attitude questionnaire consisted of 10 items assessing perceptions of GC and its screening. Items included beliefs about GC prevention, curability at early stages, the benefits of early detection, previous screening history, and reasons for not undergoing screening (e.g., lack of awareness, fear, time constraints, absence of symptoms, and financial issues). All 10 items were rated on a five‐point Likert scale (1 = strongly disagree to 5 = strongly agree), with total scores ranging from 10 to 50. Higher scores indicated more positive attitudes toward GC screening [14]. The GCHLQ consists of 24 items and was adapted from previously validated HL and cancer awareness tools [30]. Some items were modified for cultural relevance in Iran. Face and content validity were assessed by an expert panel (*N* = 10), including three oncologists, a radiologist, and six health education specialists. Items with a CVR below 0.62 were eliminated based on Lawshe's criteria [5].

They evaluated the relevance of the items and calculated the content validity ratio (CVR). The experts were also asked to determine the content validity index (CVI). Hence, we calculated CVR = 0.79 and CVI = 0.82 [6]. Additionally, a pilot study was conducted with 30 individuals to assess clarity and reliability, yielding a Cronbach's alpha (*α* = 0.79), indicating acceptable internal consistency.

The GCHLQ is a 24‐item scale designed to measure GCHL among participants. Items are rated on a five‐point Likert scale (Always, Most of the time, Sometimes, Rarely, Never) where 1 represents the lowest and 5 the highest frequency or ability.

The GCHLQ consists of the following dimensions: access (4 items, score range: 4–20), understanding and comprehension (5 items, score range: 5–25), reading (4 items, 4–20), appraisal (5 items, 5–25), and decision‐making (6 items, 6–30). The total score is calculated by summing all item scores, yielding a total score ranging from 24 to 120. Higher scores reflect higher levels of GCHL. In the absence of established clinical or population‐specific thresholds, GCHL levels were classified as low (< 31), moderate [31, 32, 33, 34, 35, 36, 37, 38, 39], and high (≥ 40) based on thresholds approximated from the sample's mean and standard deviation (35.04 ± 6.85), consistent with approaches used in similar HL studies [30]. Participants completed the questionnaire independently. Written informed consent was obtained from all participants to ensure adherence to research ethics.

Data were analyzed using independent samples *t*‐tests, one‐way ANOVA, the Pearson correlation coefficient, and multiple linear regression with SPSS software version 25. Given our conceptualization of GCHL as a multidimensional outcome influenced by sociodemographic factors, knowledge, and attitude, we used multiple linear regression with the total GCHL score as the dependent variable to assess independent associations while controlling for potential confounders. A significance level of *p* < 0.05 was considered statistically significant. The study was reported according to the STROBE (Strengthening the Reporting of Observational Studies in Epidemiology) guidelines for cross‐sectional studies [39].

### 2.1 Ethical Consideration

This study was approved by the Ethics Committee of Torbat Heydariyeh University of Medical Sciences (ID: IR.THUM.REC. 1400.038, 2021.12.27) and conducted in accordance with the Declaration of Helsinki. Participation was voluntary, with the right to withdraw at any time without penalty. Written informed consent was obtained from all participants. Data were kept confidential and used solely for research purposes. Participants showing symptoms suggestive of GC were referred for medical evaluation. All participants received brief health lifestyle promotion including recommendations related to nutrition, physical activity, and avoidance of known risk factors.

## 3 Results

The present cross‐sectional study included 304 healthy adults, with no questionnaires excluded due to missing or inconsistent responses; thus, sensitivity analyses were not required.

The mean age of the participants was 44.63 ± 11.92 years. Most were married (71.72%), and 34.87% had a college education. The majority of participants (86.52%) reported no family history of GC. 98.02% had never undergone GCS. Based on the scoring criteria, 22.5% of participants were classified as having low GCHL. The sociodemographic characteristics are presented in Table 1.

**TABLE 1:** Participant characteristics (*n* = 304).

Variable	Categories	*N* (%)
Gender	Male	96 (31.57)
	Female	208 (68.43)
Marital status	Married	218 (71.72)
	Single	86 (28.28)
Education level	Elementary education	(44.73)136
	Secondary education	29 (9.54)
	High school and a diploma	33 (10.86)
	College education	(34.87) 106
Occupational status	Housewife	(43.40) 132
	Employee	(23.01) 70
	Unemployed	36 (11.80)
	Self‐employment	66 (21.70)
Positive family or friends history of gastric cancer	Yes	41 (13.48)
	No	263 (86.52)
Experienced any lower gastrointestinal bleeding in the past month	Yes	8 (2.63)
	No	293 (96.38)
	I do not know	3 (0.98)
Experienced constipation in the past month	Yes	39 (12.82)
	No	262 (86.19)
	I do not know	3 (0.98)
Positive history of diarrhea, abdominal pain, and a feeling of fullness in the anus after defecation in the last month	Yes	56 (18.42)
	No	(78.94)240
	I do not know	8 (2.63)
Lost more than 10% of body weight during the last 6 months	Yes	21 (6.90)
	No	(93.09)283
Having a history of gastric cancer screening	Yes	6 (1.98)
	No	298 (98.02)
Total knowledge level (22 items)	Low (0–7)	108 (35.50)
	Moderate (8–14)	123 (40.50)
	High (15–22)	73 (24.00)
Why do you not undergo gastric cancer screening? multiple selections possible^a^	No symptoms	191 (62.82)
	No items	110 (36.18)
	Do not know the benefits of screening	230 (75.65)
	Financial limitations	273 (89.80)
	Fear of undergoing gastroscopy	115 (37.8)
	Worried about screening results	118 (38.8)
GCHL level	Low (< 30)	68 (22.4)
	Moderate (30–40)	186 (61.2)
	High (> 40)	50 (16.4)

Abbreviations: GC: gastric cancer, GCHL: gastric cancer health literacy.

^a^ Participants who did not undergo gastric cancer screening.

The mean scores for knowledge, attitude, and GCHL were 10.05 ± 5.48, 23.14 ± 3.84, and 35.04 ± 6.85, respectively. Pearson's correlation coefficient revealed a negative correlation between age and GCHL scores (*r* = −0.23, *p* < 0.001).

Independent samples *t*‐tests indicated no significant gender differences in knowledge (*p* = 0.58) or attitude (*p* = 0.61). However, females had significantly higher GCHL scores than males (*p* = 0.002). Marital status showed no significant association with knowledge (*p* = 0.72) or GCHL (*p* = 0.386), although single individuals had slightly higher attitude scores (*p* = 0.04). ANOVA results showed significant differences in knowledge (*p* = 0.004) and GCHL (*p* < 0.001) across education levels. Employed individuals had significantly higher knowledge and GCHL scores than unemployed participants (both *p* < 0.001). Meanwhile, the ANOVA test indicated there was no association between attitude and occupational status (*p* = 0.65). The results of the independent *t*‐test showed that participants with a family or friend history of GC had significantly higher knowledge and attitude scores (*p* < 0.001), as well as higher GCHL scores (*p* = 0.02). As shown in Table 2.

**TABLE 2:** Socio‐demographic characteristics and the mean of knowledge, attitude, and gastric cancer literacy.

Variables	Knowledge	Attitude	GCHL
	Mean ± SD	Mean ± SD	Mean ± SD
Gender			
Male	12.53 ± 2.97	23.50 ± 3.79	34.20 **±** 5.44
Female	12.37 ± 2.58	23.28 ± 4.03	36.57 ± 6.27
*P*‐value^a^	*P* = 0.58	*P* = 0.61	*P* = 0.002*
Marital status			
Married	12.93 ± 2.97	24.70 ± 2.79	35.97 ± 6.20
Single	12.77 ± 2.58	25.38 ± 1.03	34.10 ± 3.24
Widow	12.17 ± 2.58	23.70 ± 3.06	35.00 ± 10.86
Divorced	12.12 ± 2.38	20.70 ± 5.06	31.00 ± 7.00
*P*‐value^b^	*P* = 0.72	*P* = 0.04*	*P* = 0.386
Educational level			
Middle and lower	11.80 ± 2.14	27.323 ± 3.60	45.05 ± 9.15
High school and diploma	12.58 ± 2.74	27.64 ± 3.03	48.79 ± 10.06
College education	13.46 ± 2.30	27.13 ± 4.29	53.08 ± 9.70
*P*‐value^b^	*P* = 0.004*	*p* = 0.81	*p* < 0.001*
Occupational status			
Housewife	12.34 ± 2.64	27.34 ± 3.54	36.3 ± 5.81
Employee	14.59 ± 3.05	27.86 ± 4.16	37.70 ± 2.88
Unemployed	13.31 ± 2.68	26.81 ± 4.18	34.84 ± 3.88
Self‐employment	11.55 ± 2.18	27.07 ± 3.88	33.72 ± 5.84
*P*‐value^b^	*p* < 0.001*	*P* = 0.65	*p* < 0.001*
Family history of gastric cancer			
Yes	14.59 ± 3.05	27.07 ± 3.88	36.01 ± 5.81
No	12.34 ± 2.64	23.70 ± 3.06	34.03 ± 5.88
*P*‐value^a^	*p* < 0.001*	*p* < 0.001*	*P* = 0.02*

^a^ Independent *t*_ test.

^b^ One way ANOVA.

* *p* < 0.05.

A multiple linear regression was conducted to examine the association between sociodemographic factors, family history, knowledge, attitude, and the GCHL. variation The model was statistically significant, *F*(8, 295) = 39.006, *p* < 0.001; *R* ^2^ = 0.514, adjusted *R* ^2^ = 0.501. Family history had the strongest positive association with GCHL (*B* = 12.593, *β* = 0.635, *p* < 0.001), indicating that individuals with a positive family history scored, on average, 12.59 units higher on the outcome measure compared to those without such history. Knowledge score was also a significant positive predictor (*B* = 0.164, *β* = 0.131, *p* = 0.005), suggesting that higher knowledge levels are associated with better outcomes. Female gender was also significantly associated with higher GCHL (*B* = 1.569, *β* = 0.107, *p* = 0.037). Other sociodemographic variables—including age, marital status, education level, and occupational status—were not statistically significant predictors of GCHL (*p* > 0.05). Notably, attitude did not contribute significantly to the model (*p* = 0.384), despite theoretical expectations.

It is important to note that GCHL, as measured in this study, encompasses domains such as accessing, understanding, appraising, and applying health information—constructs that inherently overlap with knowledge and attitude. Consequently, although knowledge score showed a statistically significant positive association with GCHL in an exploratory model (*B* = 0.164, *p* = 0.005), this relationship should be interpreted with caution and not as evidence of a causal effect. Attitude score was not significantly associated with GCHL in the multivariable model (*p* = 0.384) (Table 3).

**TABLE 3:** Multivariable linear regression model of sociodemographic variables, family history, knowledge, and attitude in relation to GCHL.

Predictor	B	SE	*ß*	*t*	*p*
Constant	26.871	3.301	—	8.140	< 0.001
Age	0.001	0.030	0.002	0.036	0.971
Sex (1 = Female)	1.569	0.748	0.107	2.098	0.037*
Marital status (1 = Married)	−0.253	0.709	−0.016	−0.357	0.721
Education level	0.631	0.327	0.107	1.929	0.055
Occupational status	−0.126	0.260	−0.024	−0.487	0.627
Family history of gastric cancer	12.593	0.832	0.635	15.129	< 0.001***
Knowledge score	0.164	0.058	0.131	2.802	0.005**
Attitude score	0.066	0.075	0.037	0.872	0.384

*Note:* Reference groups: Male for sex; Unmarried for marital status. Model summary: *R* = 0.717, *R* ^2^ = 0.514, Adjusted *R* ^2^ = 0.501, *F*(8, 295) = 39.006, *p* < 0.001. Significance levels: *p < 0.05, **p < 0.01, ***p < 0.001.

Abbreviations: β = standardized regression coefficient, B = unstandardized regression coefficient, GCHL = gastric cancer health.

**FIGURE 1:**
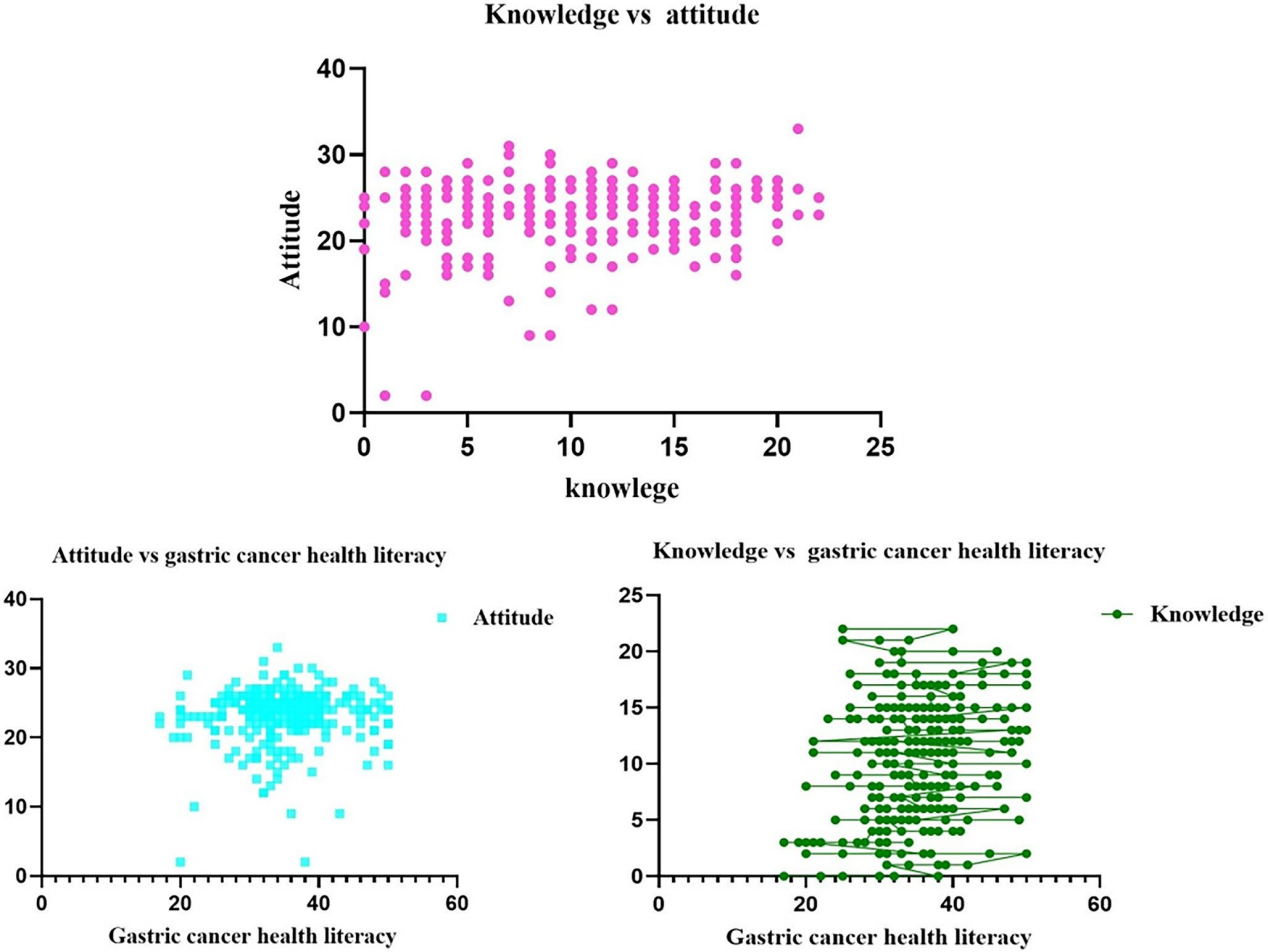
Correlation between knowledge and attitude toward gastric cancer health literacy. Scatter plots showing correlations among knowledge, attitude, and gastric cancer health literacy (GCHL) scores among participants (*N* = 304). The top plot displays the relationship between knowledge and attitude. The bottom left plot shows the relationship between attitude and gastric cancer health literacy, while the bottom right plot depicts the relationship between knowledge and gastric cancer health literacy.

Bivariate correlations further revealed a positive association between knowledge and attitude toward GC (*r* = 0.16, *p* < 0.001), a moderate correlation between knowledge and GCHL (*r* = 0.30, *p* < 0.001), and a weak but statistically significant correlation between attitude and GCHL (*r* = 0.13, *p* = 0.03). Given the conceptual overlap among these constructs, these correlations likely reflect shared variance rather than directional or causal relationships (Figure 1).

**FIGURE 2:**
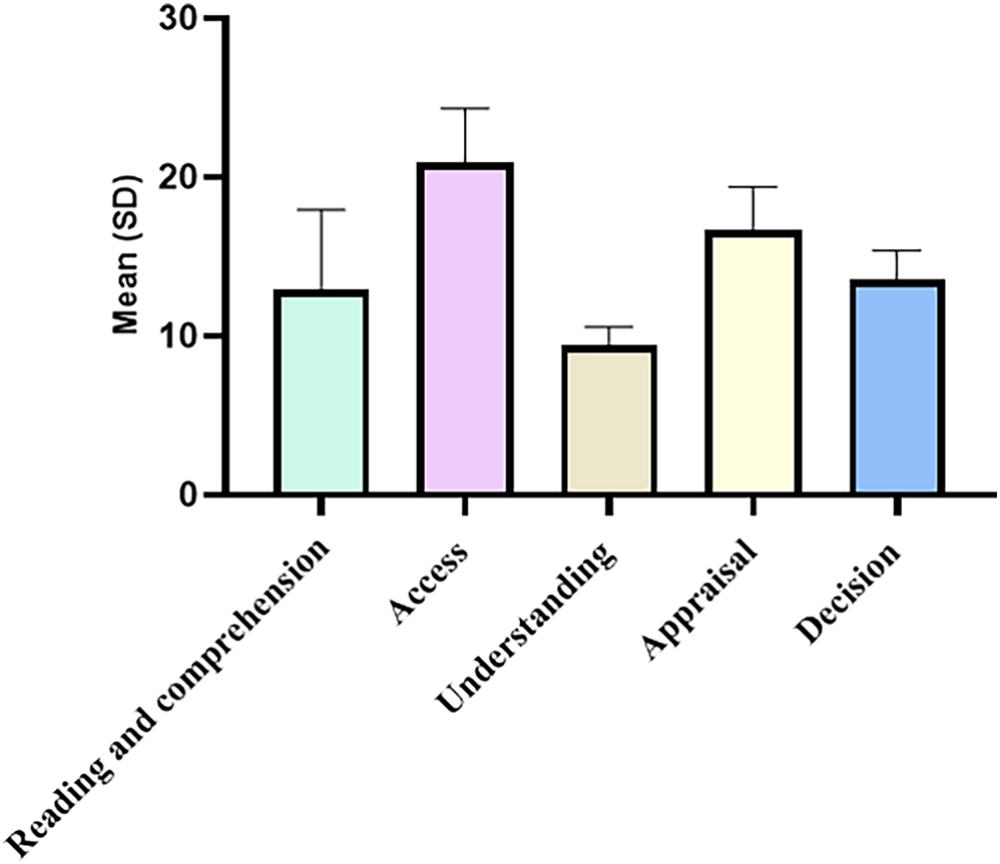
Distribution of gastric cancer health literacy dimensions. Mean score of gastric cancer health literacy (GCHL) dimensions: access, understanding, appraisal, and decision‐making (*N* = 304). Scores are presented with standard deviations. The understanding domain had the lowest mean score, whereas access, appraisal, and decision‐making domains were in the moderate range.

Figure 2 shows mean scores across GCHL domains. The understanding domain had the lowest mean score, whereas access, appraisal, and decision‐making domains were in the moderate range.

## 4 Discussion

This study assessed GCHL and its associated factors among a sample of the population in northeastern Iran. Although more than half of the participants expressed favorable attitudes toward GCS, most lacked adequate knowledge of GC risk factors and early symptoms. This knowledge gap may contribute to delays in diagnosis, potentially resulting in more advanced disease stages at the time of presentation—an observation consistent with previous studies [14, 33]. Compared to populations in China [38] and Korea [35], our participants demonstrated markedly lower levels of GC‐related knowledge. Notably, although a positive correlation was found between knowledge, attitudes, and GCHL, the cross‐sectional nature of this study precludes any conclusions about causality.

Future longitudinal studies are warranted to determine whether knowledge translates into better screening practices and earlier detection. Sociodemographic factors such as age, education, and employment status were significantly associated with GCHL. The inverse relationship between age and GCHL is particularly concerning, given the rising incidence of GC with age [36]. Middle‐aged and older adults, therefore, represent a crucial target group for tailored educational and screening interventions. Interestingly, although women had higher overall GCHL scores—consistent with studies in Denmark [34] and Korea [37]—no significant gender differences were observed in the knowledge subdomain. This contrasts with previous findings suggesting that women are generally more informed about GC [22, 38]. Women's increased engagement with healthcare services may partly explain this discrepancy [22].

### 4.1 Barriers to Screening

Participants frequently cited emotional (fear, fatalism) and economic (e.g., cost) barriers to GCS, a pattern corroborated by previous studies [2, 23, 24]. In Iran, GCS is largely opportunistic, relying on individuals to both initiate and finance the screening process themselves. Consequently, the screening rate remains extremely low (1.98%). This result underscores the pressing need for policies that reduce structural and psychological barriers to GCS uptake [10, 12].

### 4.2 Role of Education and Employment

Participants with higher education levels showed significantly better GC‐related knowledge and GCHL scores. This trend aligns with studies by Al‐Shafar et al. [31] and Almoajel et al. [18], Gondern Cakmak and Uncu [22], though it differs from findings by Christou et al. [32]. A strong positive correlation between educational attainment and GCHL was also observed. Supporting this, Jiaxuan et al. [28] reported that better survival outcomes among more educated GC patients. These findings underscore the role of education as a key determinant in both the prevention and management of GC.

Employment status was also a significant predictor: unemployed individuals had significantly lower knowledge and GCHL scores, while employed participants demonstrated higher scores in GCHL. These results are consistent with research in Denmark by Svendsen et al. [40], which found that individuals receiving unemployment or social benefits were more likely to have inadequate HL.

### 4.3 Regression Findings and Implications

The multivariate regression model explained 51.4% of the variance in GCHL. In this model, family history of GC, knowledge score, and female gender were significantly associated with higher GCHL scores.

The primary finding indicates that healthy individuals with a family history of GC reported significantly higher levels of GCHL, which may reflect greater awareness or increased attention to GC–related health information in this at‐risk subgroup. Notably, having a family history of cancer was associated with more active engagement in health information‐seeking—a pattern consistent with prior studies [33, 41]. Given this heightened attentiveness, individuals with a family history may be particularly receptive to tailored health communication strategies. Such engagement has been previously linked to higher levels of health literacy [36].

The unexpected finding of no significant association between attitude and GCHL may indicate a gap between subjective beliefs and actual health behaviors. In other words, individuals may have a positive attitude toward GC prevention, but this attitude may not necessarily lead to increased HL or action—especially in the absence of access to adequate information or environmental support. In contrast, family cancer history, as a threatening and personal experience, may provide a stronger motivation to seek information, increase knowledge, and, consequently, improve HL [42].

### 4.4 Low Screening Uptake: A Missed Opportunity

Only 1.98% of participants had ever undergone GCS, reflecting a serious gap in early detection. This figure is substantially below the rates reported in countries with national screening programs. For example, Korea's GCS coverage rose from 39.2% in 2004 to 77.5% in 2023 (an average annual increase of 3.50%) [40]. Similarly, a study in China reported that the average participation rate in upper gastrointestinal (UGI) endoscopic screening was 26.07%. In contrast, the extremely low rate observed in this study indicates not only a lack of organized national programs in Iran but also highlights gaps in public awareness, accessibility of diagnostic services, and health system prioritization. Moreover, qualitative studies from Iran suggest that public knowledge about GC risk factors and symptoms remains limited, and that primary care providers may not consistently refer high‐risk individuals for screening.

Iran's northern provinces have relatively high GC incidence. The absence of structured screening programs in high‐incidence provinces in Iran likely contributes to late‐stage diagnoses and poor prognosis.

Most participants had never undergone a gastroscopy, likely due to limited HL, low awareness, or restricted access to screening services [43]. Therefore, identifying key obstacles to GCS in Iran is essential. Since HL is related to CS behaviors [17, 19, 32], and given the high incidence of GC in Iran, pilot interventions focused on HL or community‐based screening may be considered. Further research is needed to evaluate their feasibility and effectiveness before large‐scale implementation. Accordingly, this study can serve as a foundation for evaluating the impact of future health promotion campaigns.

Qualitative studies further suggest that primary care physicians rarely refer high‐risk individuals for screening due to limited awareness or system inefficiencies.

Given the observed deficiencies in GCHL, and acknowledging the cross‐sectional and localized nature of this study, we recommend caution, small‐scale pilot interventions rather than nationwide policy changes at this stage; efforts might focus on:

Developing culturally appropriate, internet‐based health education programs. Culturally adapted HL education programs and evaluating their feasibility before broader integration within the national healthcare system [44].

1. Adapting effective international strategies for local use.
2. Pilot‐testing community‐based HL interventions in high‐incidence areas.
3. Utilizing digital platforms (e.g., mobile applications, websites, and social media) to improve outreach.
4. Engaging trusted community stakeholders, including religious leaders and local organizations.

## 5 Limitations

This study has several limitations. First, it relies on self‐reported data, which may introduce recall bias. Second, participants were recruited exclusively from CHSCs, which may have over‐represented health‐conscious individuals. Additionally, the number of participants raises concerns about potential selection bias. Individuals who seek services from such facilities may be more health‐conscious, possess better HL, or demonstrate more proactive attitudes toward health, compared to the general population. As a result, our sample may not be fully representative of broader community segments, especially those who do not regularly access healthcare services. To improve representativeness, future studies should consider using sample methods or conducting home‐based surveys. Another limitation of the study was its cross‐sectional design which prevents inference about causal relationships between variables. Moreover, we did not explore other potential influences, such as health beliefs, cultural norms, or cancer risk perception, which may impact HL, knowledge, and attitude related to GC.

Despite these limitations, the current findings still provide valuable insights into the determinants of GCHL, a health‐seeking population. Our findings may serve as a reference for other countries or cultures with high GC incidence and limited screening.

Considering the significantly low and moderate levels of GCHL observed, identifying contributing factors may offer initial insights for the development of targeted health interventions. HL plays a crucial role in individuals' understanding of preventive behaviors related to GC. It has been linked in previous research to improved health outcomes, more effective communication between patients and providers, and better utilization of healthcare services. However, these associations should be interpreted with caution, given the study's cross‐sectional design and limited geographic scope.

## 6 Conclusions

This study identified moderate levels of GCHL among clients of CHCs in northeastern Iran. Enhancing GCHL may facilitate earlier symptom recognition and promote access to diagnostic services. Longitudinal and interventional studies are necessary to clarify the causal pathways and evaluate the effectiveness of targeted strategies. Qualitative research exploring socio‐cultural and structural determinants of GCHL is therefore recommended.

Although previous studies have associated low HL with poorer health outcomes, higher health care costs, and increased hospitalization rates [22, 45], these outcomes, however, were not assessed in this study. Moreover, the suggested link between low GCHL and delayed diagnosis or poorer survival outcomes remains hypothetical and would require longitudinal or outcome‐based studies for validation.

## Author Contributions

**Kianoosh Yavarmanesh:** investigation, software, visualization, data curation, writing – original draft, writing – review and editing.

## Funding

The study was financially supported by Torbat Heydariyeh University of Medical Sciences (IR.THUM.REC. 1400.038, 2021.12.27).

## Conflicts of Interest

The authors declare no conflicts of interest.

## Data Availability

The data that support the findings of this study are available on request from the corresponding author. The data are not publicly available due to privacy or ethical restrictions.
